# Prosthesis Position after TAVI with Balloon-Expandable SAPIEN 3 in Bicuspid Aortic Valves

**DOI:** 10.3390/jcm10122561

**Published:** 2021-06-09

**Authors:** Philipp Breitbart, Jan Minners, Martin Czerny, Manuel Hein, Franz-Josef Neumann, Philipp Ruile

**Affiliations:** 1 Division of Cardiology & Angiology II, University Heart Center Freiburg • Bad Krozingen, University Hospital Freiburg, 79189 Bad Krozingen, Germany; Jan.Minners@universitaets-herzzentrum.de (J.M.); Manuel.Hein@universitaets-herzzentrum.de (M.H.); Franz-Josef.Neumann@universitaets-herzzentrum.de (F.-J.N.); Philipp.Ruile@universitaets-herzzentrum.de (P.R.); 2 Department of Cardiovascular Surgery, University Heart Center Freiburg • Bad Krozingen, University Hospital Freiburg, 79189 Bad Krozingen, Germany; martin.czerny@universitaets-herzzentrum.de

**Keywords:** transcatheter aortic valve implantation, TAVI, bicuspid aortic valves, prosthesis positioning, computed tomography angiography, fusion imaging

## Abstract

Background: Prior data suggest a correlation between the position of transcatheter heart valves (THV) and the occurrence of complications after transcatheter aortic valve implantation (TAVI) in patients with tricuspid aortic valves (TAV). However, data including a detailed analysis of prosthesis positioning in bicuspid aortic valves (BAV) are limited. Therefore, the purpose of this study was to investigate THV position after TAVI in BAV. Methods: We evaluated the THV position in 50 BAV and 50 TAV patients (all received the balloon-expandable Sapien 3 prosthesis) using fusion imaging of pre- and post-procedural computed tomography angiography. According to the manufacturers’ recommendations, a low implantation position was defined as >30% of the prosthesis below the annulus. Results: THV position was appropriate in the majority of the patients within both groups (90.0% for BAV vs. 96.0% for TAV, *p* = 0.240). In BAV, we observed a more pronounced THV waist (7.4 ± 4.5% vs. 5.8 ± 3.0%, *p* = 0.043) and a lower average THV expansion (91.9 ± 12.2% vs. 95.5 ± 2.7% of nominal expansion, *p* = 0.044). Conclusions: Accurate positioning in relation to the aortic annulus of the TAVI Sapien 3 prosthesis is possible in patients with BAV with results comparable to TAV. However, there is a more pronounced prosthesis waist and a lower average THV expansion in BAV.

## 1. Introduction

Bicuspid aortic valves (BAVs) are the most common congenital heart defect, with an overall incidence of 1–2%, and are responsible for over 50% of symptomatic aortic valve stenoses in patients < 80 years [1]. Nevertheless, up to 20% of elderly patients considered for transcatheter aortic valve implantation (TAVI) exhibit bicuspid valves [2]. However, most early major randomized trials investigating the TAVI procedure excluded BAV [3,4].

An early study with a limited number of patients demonstrated the TAVI feasibility in BAV-patients with encouraging short- and intermediate-term clinical outcomes but questioned the long-term outcome due to suboptimal echocardiographic results [5]. Current data suggest that TAVI in BAV is safe and effective, especially in newer generations of prosthesis designs with improved outcomes comparable to those of tricuspid aortic valves (TAVs) [6,7,8,9,10]. Current large clinical trials included a proportion of 2–6% of patients with BAV [11,12]. Asymmetric valve geometry may render prosthesis positioning difficult in clinical practice, leading to transcatheter heart valve (THV) malpositioning. This may lead to various complications, e.g., conduction disturbances or paravalvular leakage [13,14]. While THV position and characteristics were mostly examined in patients with TAV, data for TAVI in BAV are scarce so far. A prior study of our group demonstrated an exact three-dimensional visualization of the THV within the native annulus region after TAVI using a new fusion imaging method of pre- and post-procedural computed tomography angiography (CTA) [15]. With this method, we revealed deep implantation of the THV as a predictor for the new onset of conduction disturbances.

Therefore, this study aimed to investigate the THV position after TAVI in BAV compared to TAV using this fusion imaging method.

## 2. Materials and Methods

### 2.1. Study Population

Patients with an analyzable pre- and post-TAVI CTA and implanted Sapien 3 THV (Edwards Lifesciences Inc., Irvine, CA, USA) were included in this retrospective single-center study. In our institution, all eligible patients receive routine post-TAVI CTAs according to guidelines concerning thoracic aortic stent implantation [16], with the intention to identify possible (subclinical) complications, e.g., aortic injuries or thrombosis of the valves. Severe renal insufficiency, frailty, and others were contraindications for a post-procedural CTA [17].

Within the study population, 50 patients with a BAV were diagnosed in pre-TAVI CTA. These patients were compared with a 1:1-matched (regarding prosthesis size with 23 mm, 26 mm, and 29 mm Sapien 3) TAV control group. The study was approved by the local institutional review board (IRB number EF FR 472/12) and complies with the Declaration of Helsinki.

### 2.2. Morphology of the Bicuspid Aortic Valves

**Figure 1:**
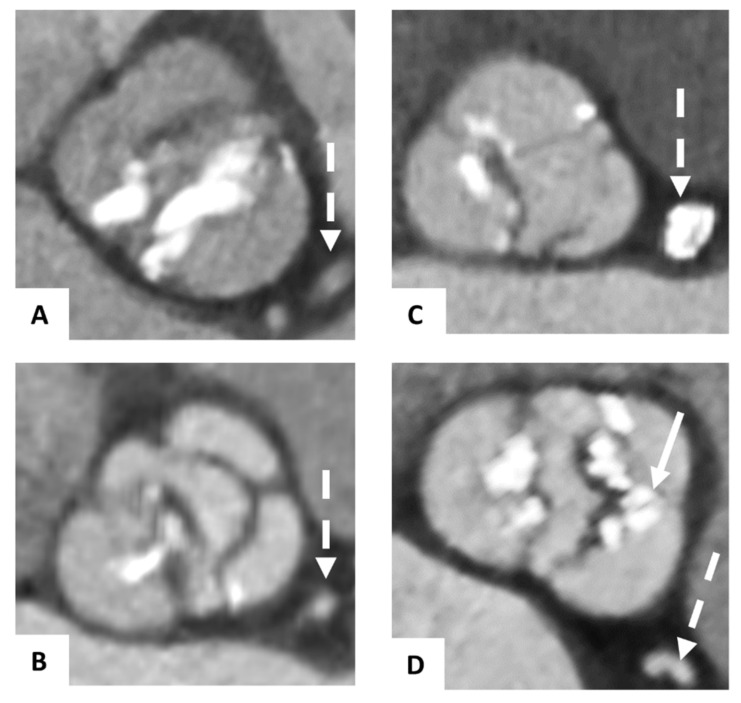
Classification of bicuspid aortic valves. Pre-TAVI contrast-enhanced CTA axial showing congenital bicuspid aortic valves classified according to the schema of Sievers and Schmidke with Type 0 (**A**), Type 1 L/R (with raphe between the left and right coronary cusps) (**B**), Type 1 R/N (with raphe between the right and non-coronary cusps), (**C**) and a functional bicuspid aortic valve (**D**) with secondarily fused left and right coronary cusps due to severe calcification (→). The dashed arrow (--->) is marking the left coronary artery system.

The morphology of the BAVs was classified in pre-TAVI CTA according to the scheme of Sievers and Schmidke [18]. The congenital BAVs were divided into 3 major types, depending on the number of cusps and the presence of raphes: 0 (without any raphes), 1 (one raphe), or 2 (two raphes) (Figure 1). Type 1 was subclassified regarding the localization of the raphe in L/R with a raphe between the left (L) and right (R) coronary cusps, L/N with raphe between the left and non-coronary (N) cusps, or R/N. As previously described, we defined functional (acquired) as secondarily fused cusps due to the adhesion of the commissure between two cusps, presumably due to degenerative processes [7].

### 2.3. Image Acquisition

We performed retrospective ECG-gated contrast-enhanced pre- and post-TAVI CTAs (70 mL for pre- and 50 mL for post-TAVI CTA, Imeron 400, Bracco, Konstanz, Germany) with a second-generation, dual-source CT scanner (Somatom Definition Flash, Siemens Healthineers, Forchheim, Germany) with previously described CTA-protocols [17]. All post-TAVI CTAs were performed before discharge.

We used bolus tracking within the left atrium as the region of interest for beginning the scan. Reconstruction of images was conducted in 50 ms steps throughout the cardiac cycle; slice thickness was 1 mm with an increment of 0.8 mm, applying a stent-specific reconstruction kernel B46f for post-TAVI CTA.

Images based on multiplanar reformations were analyzed at a post-processing workstation (Syngo Multimodality Workplace, Siemens Healthineers, Forchheim, Germany).

### 2.4. Process of Fusion Imaging

**Figure 2:**
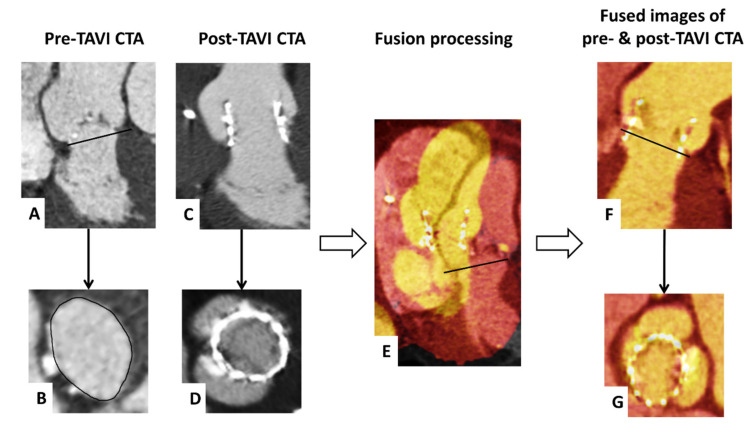
Visualization of fusion imaging of pre- and post-TAVI CTA. CTA axial and sagittal oblique reconstructions show the pre-TAVI images with delineating of the annulus plane (**A**,**B**) and post-TAVI images with the implanted Sapien 3 (**C**,**D**). Subsequently, the pre- and post-TAVI images were semi-automatically merged (**E**). Finally, the fused images were manually adapted to achieve an optimal alignment of the annular region in all reconstructions (**F**,**G**).

We assessed the final prosthesis position with fused images of pre- and post-procedural CTA, as previously described by our group [15]. In brief, we defined the annulus plane within the pre-TAVI CTA followed by a semi-automatic merging of these images with the post-TAVI CTA at the corresponding reconstruction time-point during systole. In the end, we performed a manual adaption of the fused images to achieve an optimal alignment of the annular region with their adjacent structures (Figure 2).

### 2.5. Image Analysis

**Figure 3:**
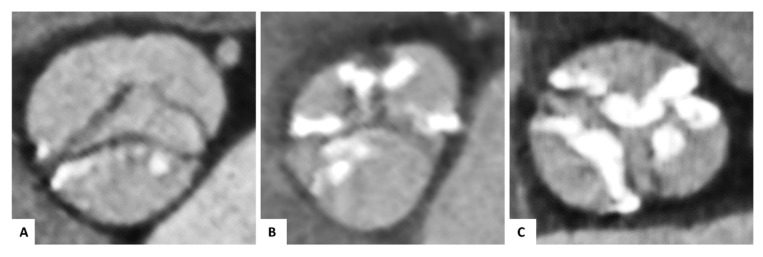
Semiquantitative calcification assessment of the device-landing zone. Pre-TAVI contrast-enhanced CTA of congenital and functional bicuspid aortic valves for visual calcification assessment with examples for mild (**A**), moderate (**B**), or severe (**C**) calcification of the cusps regions.

Each image analysis was performed by two experienced readers (P.B. and P.R.). We conducted the following measurements on pre-TAVI images: within systole, the aortic annulus area and the area derived diameter were determined, and the annulus eccentricity was calculated as the largest diameter/smallest diameter. For each cusp, we performed a semiquantitative calcification assessment of the device-landing zone (grade 0: no calcification, grade 1: mild calcification as small calcified spots with a minimal diameter ≤ 2 mm, grade 2: moderate calcification as calcified spots with a minimal diameter more than 2 mm, grade 3: severe calcification as large calcified formations more than 5 mm minimal diameter), as previously described (Figure 3) [19]. Measurements of total THV length, THV distance above and below the annulus (separately for the left coronary cusp, right coronary cusp, and non-coronary cusp) were conducted within the fused images to analyze the implantation depth. In the case of BAV without any raphes, we performed the measurements for the left coronary cusp adjacent to the ostium of the left coronary artery; for the right coronary cusp, adjacent to the ostium of the right coronary artery; for the non-coronary cusp, opposite of the middle between the left coronary and right coronary cusp. We defined the optimal THV position according to the manufacturers’ recommendations as more than 70 percent of the prosthesis above (aortic) and less than 30 percent below (ventricular) the annulus plane.

The arctangent of (maximum–minimum stent center height above the annulus plane)/(mean expanded THV diameter × 180/Pi) defined the THV tilt in relation to the annulus plane. We assessed the expanded THV area using the mean value to evaluate the average prosthesis expansion as a percentage of the nominal area on three different heights (left ventricular outflow tract end, center of the stent, aortic end). The prosthesis oversizing (%) was determined as ((manufacturer-reported THV area/mean annulus area-1) × 100). For definition of the extent of prosthesis waist in percent area, measurements of the stent center compared to the average of the stent entry and exit were utilized.

### 2.6. Statistical Analysis

We performed all statistical analyses with SPSS software, Version 25.0 (IBM Corp., Armonk, NY, USA), reporting categorical data as frequencies or percentages and continuous variables as a mean with standard deviation or median with interquartile range. The χ2-test (for categorical variables), Student’s *t*-test (for normally distributed continuous variables), Mann–Whitney-U test (non-normally distributed continuous variables in the comparison of BAVs with the control group), or Kruskal–Wallis test (non-normally distributed continuous variables in the comparison among the BAV subtypes) was used to test differences between the BAV and control group. Continuous variables were tested for normal distribution applying the Kolmogorov–Smirnov test. We defined a *p*-value < 0.05 as statistically significant in all tests.

## 3. Results

During a period of 6 years (2014–2020), a post-TAVI CTA was conducted in 608 patients after implantation of a Sapien 3 THV within a median of 5 [interquartile range of 4–6] days after the procedure. Eleven post-TAVI CTAs were non-diagnostic for performing fusion imaging and THV position measurements due to reduced image quality. In 50 of these patients, a BAV was diagnosed. They were compared to 50 of a 1:1-matched control group as a randomized selection of the remaining 547 patients with TAV. The baseline characteristics of patients with BAV and the matched control group are presented in Table 1. Among the baseline variables, patients with BAV were significant younger (78.3 ± 5.7 vs. 81.8 ± 4.1 years, *p* = 0.001), whereas there were no significant differences regarding female gender (42.0 vs. 38.0%, *p* = 0.683), logistic Euroscore (15.5 ± 13.3% vs. 14.2 ± 9.5%, *p* = 0.941), or atrial fibrillation (26.0 vs. 32.0%, *p* = 0.509). However, patients with BAV had a higher calcification grade of the device-landing zone (5.0 ± 1.1 vs. 4.4 ± 1.3, *p* = 0.019). No major complications (death, stroke, myocardial infarction, major bleeding, contained/uncontained annular rupture, or THV embolization) occurred in both groups within the in-hospital period immediately after the procedure.

### 3.1. Procedural- and Prosthesis—Related Characteristics

Implanted prosthesis sizes were 23 mm in 14 (14%) patients, 26 mm in 54 (54%), and 29 mm in 32 (32%). The procedural- and prosthesis-related characteristics of both groups are also presented in Table 1. The rate of post-dilatation was 12.0% in both groups (*p* = 1.00). The analysis of oversizing or THV tilt revealed no significant differences between groups (*p* = 0.212 and *p* = 0.436). However, there was a more pronounced waist (7.4 ± 4.5% vs. 5.8 ± 3.0%, *p* = 0.043) and a lower average expansion (91.9 ± 12.2% vs. 95.5 ± 2.7% of nominal expansion, *p* = 0.044) in the THV in patients with BAV. Furthermore, in BAV patients, the prostheses reached more into the left ventricular outflow tract at the right coronary cusp (4.2 ± 2.9 vs. 3.3 ± 2.6 mm below annulus, *p* = 0.040). The mean implantation depth did not differ between both groups, and the majority of the THVs were implanted in an optimal position (3.8 ± 2.6 vs. 3.2 ± 2.0 mm below annulus, *p* = 0.171; 90.0% vs. 96% optimal position, *p* = 0.240). We observed no significant differences in new-onset conduction disturbances or new pacemaker implantation (*p* = 0.348 and *p* = 0.838). The amount of paravalvular THV leakage (PVL) was similar between both groups (*p* = 0.385); no patient revealed a moderate or severe PVL.

### 3.2. Subanalysis of the Bicuspid Aortic Valves

Among the BAV, 5 (10%) were classified as Sievers Type 0, 34 (68%) as Type 1 (left-right (*n* = 30); right-non (*n* = 4); left-non (*n* = 0)) and 11 (22%) as functional BAV. A separate presentation of the baseline-, procedural-, and prosthesis-related characteristics of the BAV patients, taking into account the individual bicuspid valve types, is shown in Table 2.

## 4. Discussion

To the best of our knowledge, this is the first study investigating the exact positioning of Sapien 3 THVs in bicuspid valves using fusion imaging of pre-and post-TAVI CTA within a cohort of BAV. Our data suggest that the positioning of these balloon-expandable devices in BAV shows a comparable accuracy to TAV. However, there is a more pronounced prosthesis waist and a lower average expansion of the THV in these patients.

A BAV is responsible for most isolated aortic valve replacements in younger patients but also causes stenosis in higher age groups with a proportion of up to 38% in patients between 71 and 80 years [20]. The clinical outcomes of TAVI using new-generation devices in BAV were favorable, with cumulative all-cause mortalities after 2 years comparable to TAV [21]. Prior data suggested a THV malposition as the leading cause of TAVI complications [13]. This called for a detailed analysis of THV position in patients with BAV.

### 4.1. Implantation Depth and Conduction Disturbances

The majority of THVs in BAV were implanted in an optimal position according to manufacturers’ recommendations, suggesting that TAVI with a Sapien 3 device in BAV is feasible and safe. Thus, our data confirm the hypothesis of other studies determining the THV position in post-TAVI CTA [9,22]. In these earlier trials, THV-provoked shadowing and post-interventional changes of the anatomy of the aortic root might have hampered the correct evaluation of THV positioning. In contrast, fusion imaging, as obtained in the current trial, allows for excellent visualization and assessment of the implanted THV as a three-dimensional object within the native aortic valve region [15]—the important novel findings of our study.

In view of the similar mean implantation depth between BAV and TAV, the lower prosthesis position in BAV next to the right coronary cusp needs to be interpreted cautiously. A correlation of a low prosthesis position, especially next to the right and non-coronary cusp, with new conduction disturbances post-TAVI, is reported [15,23,24]. Perlman et al. suggest that the high rate of pacemaker implantation (23.5%) in their study may be associated with difficulty in achieving exact implantation heights as a result of the irregular shape of the leaflets on fluoroscopy, possibly resulting in lower implantation [7]. In line with these data, we observed numerically more new conduction disturbances in BAV (51% vs. 41%, *p* = 0.348), yet without any appreciable impact on new pacemaker implantation. Due to the limited sample size, these observations that were far from statistical significance are difficult to interpret.

The higher amount of calcification of the device-landing zone in BAV, proven in this and prior studies, reflects the propensity for calcium deposition in these abnormally functioning valves, which is also a known predictor for new conduction disturbances and pacemaker implantations [9,25,26,27].

### 4.2. Expansion and Prosthesis Waist

We observed a lower average expansion of the THV in BAV as compared with TAV, which is in line with prior studies investigating Sapien 3 THV geometry [9,22]. This may be attributed to more pronounced calcification of the device-landing zone including calcified raphes [9]. Furthermore, we confirmed a more pronounced prosthesis waist after TAVI in BAV, previously postulated by Kawamori et al. [9]. This might be caused by forceful balloon inflation leading to dog-boning at the edges and a more restricted expansion at the annular level in the case of a BAV. Previous data from our group suggest a more pronounced prosthesis waist as protective against early leaflet thrombosis after TAVI, whereas a lower average expansion predicts this phenomenon [28]. In the light of these results, further studies with larger patient cohorts are desirable to detect possible influences of BAV on the occurrence of LT.

## 5. Limitations

This study reports on a limited patient number of 50 patients with BAV. This might hamper a diagnosis of some minor differences of the THV position or patient-specific characteristics between BAV and TAV patients, though we tried to overcome this limitation by choosing a matched control group.

## 6. Conclusions and Impact on Daily Practice

Considering the increasing application of the TAVI procedure to younger patients with a greater proportion of BAV, robust data for a reliable procedure in this subset are crucial. In this context, we demonstrate that the positioning accuracy of the balloon-expandable Sapien 3 THVs is adequate in patients with BAV.

## Figures and Tables

**Table 1:** Baseline, procedural and prosthesis-related characteristics of the entire study population, patients with bicuspid aortic valve, and the matched control group.

		All Patients (*n* = 100)	Patients with Bicuspid Valve (*n* = 50)	Matched Control Group (*n* = 50)	*p*-Value
Age	(years)	80.0 ± 5.3	78.3 ± 5.7	81.8 ± 4.1	0.001
Female		40 (40)	21 (42)	19 (38)	0.683
BMI	(kg/m^2^)	26.8 ± 4.6	26.3 ± 4.6	27.3 ± 4.6	0.061
Logistic Euroscore	(%)	14.8 ± 11.5	15.5 ± 13.3	14.2 ± 9.5	0.941
STS Score	(%)	3.8 ± 4.1	3.5 ± 2.6	4.1 ± 5.2	0.443
Previous pacemaker		9 (9)	5 (10)	4 (8)	0.727
Atrial fibrillation		29 (29)	13 (26)	16 (32)	0.509
Oral anticoagulation		15 (15)	15 (30)	15 (30)	1.000
Aortic valve area	(cm^2^)	0.78 ± 0.17	0.80 ± 0.19	0.76 ± 0.16	0.383
Annulus diameter	(mm)	25.4 ± 2.2	25.7 ± 2.4	25.2 ± 2.0	0.705
Annulus eccentricity (CTA)		1.3 ± 0.1	1.3 ± 0.1	1.3 ± 0.1	0.125
Grade of calcification of the device-landing zone	total	4.7 ± 1.2	5.0 ± 1.1	4.4 ± 1.3	0.019
	Left coronary cusp	1.5 ± 0.6	1.5 ± 0.7	1.4 ± 0.5	0.478
	Right coronary cusp	1.5 ± 0.5	1.5 ± 0.5	1.5 ± 0.5	0.821
	Non-coronary cusp	1.8 ± 0.6	2.0 ± 0.5	1.6 ± 0.5	<0.001
Ejection fraction pre-interventional	(%)	48.0 ± 10.8	46.4 ± 11.7	49.5 ± 9.8	0.262
					0.558
Access route	Transfemoral	97 (97)	49 (98)	48 (96)	
	Transapical	3 (3)	1 (2)	2 (4)	
Prosthesis size					1.000
	23 mm	14 (14)	7 (14)	7 (14)	
	26 mm	54 (54)	27 (54)	27 (54)	
	29 mm	32 (32)	16 (32)	16 (32)	
Post-dilatation		12 (12)	6 (12)	6 (12)	1.000
Underfilling		12 (12)	6 (12)	6 (12)	1.000
New CD after TAVI ^a^		42 (46.2)	23 (51.1)	19 (41.3)	0.348
New PM after TAVI ^a^		19 (20.9)	9 (20.0)	10 (21.7)	0.838
Prosthesis oversizing	(%)	7.0 ± 10.0	5.5 ± 10.3	8.5 ± 9.5	0.212
THV tilt	(°)	5.4 ± 3.4	5.6 ± 3.6	5.2 ± 3.4	0.436
Extent of the THV waist	(%)	6.6 ± 3.9	7.4 ± 4.5	5.8 ± 3.0	0.043
THV expansion	(%)	93.7 ± 9.0	91.9 ± 12.2	95.5 ± 2.7	0.044
Mean expanded THV area	(mm^2^)	466.2 ± 76.9	457.6 ± 79.7	474.9 ± 73.7	0.166
MPG after implantation	(mmHg)	11.0 ± 3.6	11.3 ± 3.9	10.7 ± 3.3	0.334
Leaflet thrombosis		16 (16)	7 (14)	9 (18)	0.585
					0.385
	None	47 (47)	22 (44)	25 (50)	
Paravalvular	Trivial	27 (27)	12 (24)	15 (30)	
leakage	Mild	26 (26)	16 (32)	10 (20)	
	Moderate	0 (0)	0 (0)	0 (0)	
	Severe	0 (0)	0 (0)	0 (0)	
	Mean	3.5 ± 2.3	3.8 ± 2.6	3.2 ± 2.0	0.171
Implantation depth below	Left coronary cusp	2.9 ± 2.6	3.2 ± 2.9	2.6 ± 2.2	0.273
annulus (mm)	Right coronary cusp	3.8 ± 2.8	4.2 ± 2.9	3.3 ± 2.6	0.040
	Non-coronary cusp	3.8 ± 2.7	4.1 ± 3.1	3.5 ± 2.3	0.607
					0.240
Prosthesis position	Optimal	93 (93)	45 (90)	48 (96)	
	Low	7 (7)	5 (10)	2 (4)	

Values are mean ± standard deviation or n (%). BMI: body mass index. CD: conduction disturbances. CTA: computed tomography angiography. MPG: mean pressure gradient. PM: permanent pacemaker. STS: Society of Thoracic Surgeons. THV: transcatheter heart valve. ^a^ Percent value based on patients without previous permanent pacemakers.

**Table 2:** Baseline, procedural, and THV position characteristics of the entire patient population with bicuspid aortic valves and for individual BAV types.

		All Patients with Bicuspid Valves (*n* = 50)	Sievers 0 (*n* = 5)	Sievers 1 (*n* = 34)	Functional (*n* = 11)	*p*-Value
Annulus diameter	(mm)	25.7 ± 2.4	27.0 ± 2.0	25.3 ± 2.2	26.2 ± 3.2	0.221
Annulus eccentricity (CTA)		1.3 ± 0.1	1.1 ± 0.2	1.3 ± 0.1	1.3 ± 0.2	0.082
Grade of calcification of the device-landing zone	total	5.0 ± 1.1	5.3 ± 0.6	5.0 ± 1.2	5.0 ± 1.0	0.677
	Left coronary cusp	1.5 ± 0.7	1.9 ± 0.8	1.5 ± 0.7	1.5 ± 0.6	0.532
	Right coronary cusp	1.5 ± 0.5	1.8 ± 0.7	1.4 ± 0.4	1.7 ± 0.5	0.089
	Non-coronary cusp	2.0 ± 0.5	1.6 ± 0.6	2.1 ± 0.5	1.8 ± 0.5	0.082
Prosthesis size						0.143
	23 mm	7 (14)	0 (0)	5 (14.7)	2 (18.2)	
	26 mm	27 (54)	1 (20)	21 (61.8)	5 (45.5)	
	29 mm	16 (32)	4 (80)	8 (23.5)	4 (36.4)	
Post-dilatation		6 (12)	3 (60)	1 (2.9)	2 (18.2)	0.001
Underfilling		6 (12)	0 (0)	5 (14.7)	1 (9.1)	0.605
Prosthesis oversizing	(%)	5.5 ± 10.3	9.4 ± 8.6	6.2 ± 9.9	1.9 ± 12.0	0.342
THV tilt	(°)	5.6 ± 3.6	4.4 ± 2.9	5.3 ± 3.7	7.0 ± 3.0	0.204
Hourglass form of the THV	(%)	7.4 ± 4.5	3.9 ± 6.4	7.4 ± 4.3	8.9 ± 3.7	0.214
THV deployment	(%)	91.9 ± 12.2	94.4 ± 3.6	90.6 ± 14.6	94.7 ± 3.4	0.421
Mean expanded THV area	(mm^2^)	457.6 ± 79.7	536.1 ± 65.2	443.3 ± 70.7	466.2 ± 95.5	0.098
MPG after implantation	(mmHg)	11.3 ± 3.9	9.8 ± 2.3	11.0 ± 3.2	13.0 ± 5.7	0.292
	Mean	3.8 ± 2.6	4.7 ± 4.2	4.0 ± 2.4	3.1 ± 2.3	0.474
Implantation depth below	Left coronary cusp	3.2 ± 2.9	4.0 ± 4.3	3.4 ± 2.7	2.4 ± 2.7	0.500
annulus (mm)	Right coronary cusp	4.2 ± 2.9	3.4 ± 4.2	4.9 ± 2.4	2.7 ± 3.5	0.130
	Non-coronary cusp	4.1 ± 3.1	6.6 ± 4.8	3.7 ± 3.0	4.1 ± 2.1	0.382
						0.734
Prosthesis position	Optimal	45 (90)	4 (80)	31 (91.2)	10 (90.9)	
	Low	5 (10)	1 (20)	3 (8.8)	1 (9.1)	

Values are mean ± standard deviation or n (%). CTA: computed tomography angiography. MPG: mean pressure gradient. THV: transcatheter heart valve.

## Data Availability

The data presented in this study are available on request from the corresponding author.

## Abbreviations

BAV	Bicuspid aortic valve
CTA	Computed tomography angiography
PVL	Paravalvular THV leakage
TAV	Tricuspid aortic valve
TAVI	Transcatheter aortic valve implantation
THV	Transcatheter heart valve
